# Deficiency of peroxiredoxin 6 or inhibition of its phospholipase A_2_ activity impair the *in vitro* sperm fertilizing competence in mice

**DOI:** 10.1038/s41598-017-13411-2

**Published:** 2017-10-11

**Authors:** Adel R. Moawad, Maria C. Fernandez, Eleonora Scarlata, Chandra Dodia, Sheldon I. Feinstein, Aron B. Fisher, Cristian O’Flaherty

**Affiliations:** 1 The Research Institute of the McGill University Health Centre, McGill University, Montréal, Québec Canada; 2 Departments of Surgery (Urology Division), McGill University, Montréal, Québec Canada; 3 Pharmacology and Therapeutics, McGill University, Montréal, Québec Canada; 4 Department of Theriogenology Faculty of Veterinary Medicine, Cairo University, Giza, Egypt; 5 Institute for Environmental Medicine, Perelman School of Medicine, University of Pennsylvania, Philadelphia, PA USA; 6 Department of Physiology, Perelman School of Medicine, University of Pennsylvania, Philadelphia, PA USA

## Abstract

*Prdx6* ^−/−^ male mice are subfertile, and the deficiency or inactivation of Peroxiredoxins (PRDXs) is associated with human male infertility. We elucidate the impact of the lack of PRDX6 or inhibition of its calcium-independent phospholipase A_2_ (Ca^2+^-iPLA_2_) activity by MJ33 on fertilization competence of mouse spermatozoa. Sperm motility, viability, fertilization and blastocyst rates were lower in *Prdx6* ^−/−^ spermatozoa than in C57BL/6J wild-type (WT) controls (*p* ≤ 0.05). MJ33 inhibited the PRDX6 Ca^2+^-iPLA_2_ activity and reduced these parameters in WT spermatozoa compared with controls (*p* ≤ 0.05). Levels of lipid peroxidation and of superoxide anion (O_2_^•─^) were higher in *Prdx6* ^−/−^ than in WT spermatozoa (*p* ≤ 0.05). MJ33 increased the levels of lipid peroxidation and mitochondrial O_2_^•─^ production in treated versus non-treated WT spermatozoa. Acrosome reaction, binding to zona pellucida and fusion with the oolemma were lower in *Prdx6* ^−/−^ capacitated spermatozoa than WT capacitated controls and lower in WT spermatozoa treated with the PRDX6 inhibitor. In conclusion, the inhibition of the PRDX6 Ca^2+^-iPLA_2_ activity promotes an oxidative stress affecting viability, motility, and the ability of mouse spermatozoa to fertilize oocytes. Thus, PRDX6 has a critical role in the protection of the mouse spermatozoon against oxidative stress to assure fertilizing competence.

## Introduction

Infertility is a common health problem among human populations; it affects approximately 15% of couples^1^ and 50% of these cases are ascribed to male factor infertility^2,3^. Various factors either pathological such as varicocele, cryptorchidism, infections, nutritional deficiencies and trauma or environmental exposure to chemicals, drugs, smoke, toxins, radiations or pollutants have been linked to perturbations occurring during spermatogenesis that lead to male infertility^4–7^. Interestingly, oxidative stress is considered a major player in the pathophysiological mechanisms in the above-mentioned conditions^5,7–10^. Oxidative stress occurs as a consequence of an imbalance between the levels of reactive oxygen species (ROS) production and the antioxidant capacity of the cell^11^. The oxidative stress-dependent damage impairs cell function and can lead to cell death^12,13^. High levels of ROS promote impairment of motility and capacitation, the processes that allow the spermatozoon to fertilize the oocyte^14^. ROS-mediated injury to the spermatozoa is the main contributing factor in up to 80% of infertile men^15^. Under these conditions, spermatozoa experience damages such as peroxidation of membrane lipids^16^, oxidation of proteins^14^, DNA fragmentation and oxidation^17,18^, low mitochondrial membrane potential^19,20^, and decreased levels of energy (ATP production) that lead to an impairment of motility^21,22^. Although excessive amounts of ROS are harmful to the sperm cells^23^, low levels of these molecules are needed to induce sperm capacitation^24–26^.

The presence of antioxidant enzymes is important to circumvent oxidative damage in the spermatozoa; however, the antioxidant protection is limited in this cell type^10,27,28^. Interestingly, glutathione peroxidase 4 (GPX4), the only GPX present in human spermatozoa, is localized in the mitochondrial sheath as an insoluble protein without antioxidant activity, and it is also present in the sperm nucleus^29^. The major sperm antioxidant enzymes appear to be the peroxiredoxin (PRDX) family and the superoxide dismutases 1 and 2^10,28^. Peroxiredoxins are selenium free peroxidases highly abundant in human spermatozoa^28,30^. The six mammalian members of the family are differentially localized in compartments of the human spermatozoon, PRDX6 being the most abundant and present in all compartments^28,30^. PRDX1-5 have two catalytically active cysteines per molecule (2-Cys PRDXs), while PRDX6 has only one. All PRDXs can reduce hydrogen peroxide, single-chain organic peroxides, and peroxynitrite^31–33^. Interestingly, PRDX6 can reduce phospholipid hydroperoxides (using glutathione) as well and has calcium-independent phospholipase A_2_ (Ca^2+^-iPLA_2_) and lysophospholipid acyltransferase activities^31,34–36^

We recently reported that seminal plasma and spermatozoa from infertile men have lower levels of PRDXs than healthy donors^27^. Furthermore, the inhibition of the peroxidase activity of 2-Cys PRDXs and of Ca^2+^-iPLA_2_ of PRDX6 prevented sperm capacitation and its associated actin polymerization^37^. The absence of PRDX6 had a negative impact on the quality of mouse spermatozoa (reduced motility, high levels of DNA and protein oxidation and abnormal sperm chromatin structure) that promote a decrease in fertility outcomes^38,39^. Moreover, this abnormal reproductive phenotype worsens as the male mice age^38^. *Prdx6* ^−/−^ male mice are susceptible to *in vivo* oxidative stress generated by tert-BHP treatment showing higher levels of DNA oxidation and lower sperm quality and capacitation than the non-treated *Prdx6* ^−/−^ controls^39^.

In view of the great abundance of PRDX6 in normal semen, its role in the protection against oxidative stress of human spermatozoa^30^, and its necessity to assure the production of normal spermatozoa, as seen in our *Prdx6* ^−/−^ mouse model^38^, the aim of this study was to elucidate the impact of the absence of PRDX6 and inhibition of its Ca^2+^-iPLA_2_ activity on different sperm functions that are essential for the fertilizing ability of the spermatozoon.

## Results

### Prdx6^−/−^ testis have normal spermatogenesis and normal sperm morphology

The histological analysis of testis tissues revealed that *Prdx6* ^−/−^ males have normal spermatogenesis (Supplementary Fig. S1A and B). *Prdx6* ^−/−^ spermatozoa have normal morphology and presence of normal acrosomes, similarly as we observed in WT spermatozoa (Supplementary Fig. S1C–F). However, despite this, as we reported before^39^, *Prdx6* ^−/−^ male mice are subfertile with their spermatozoa displaying low motility, high levels of DNA oxidation and abnormal sperm chromatin structure.

### Activity of Ca^2+^-iPLA_2_ in WT and Prdx6^−/−^ spermatozoa treated with MJ33

The activity of Ca^2+^-iPLA_2_ was dramatically decreased in MJ33-treated WT spermatozoa compared to non-treated WT controls (Table 1). These results confirm that MJ33 does inhibit the PRDX6 Ca^2+^-iPLA_2_ activity in mouse spermatozoa. The Ca^2+^-iPLA_2_ activity of *Prdx6* ^−/−^ spermatozoa was even lower than that of the MJ33-treated WT controls.

**Table 1:** MJ33 inhibits the Ca^2+^-iPLA_2_ activity of PRDX6 in mouse spermatozoa.

**Mice**	**MJ33 (µM)**	**Ca** ^**2+**^ **-iPLA**_**2**_ **activity (nmol/h per mg prot)**
***Prdx6*** ^−/−^	0	0.026 ± 0.012^a^
	10	0.007 ± 0.021^a^
	20	ND
**WT**	0	10.3 ± 0.20^b^
	10	1.3 ± 0.11^c^
	20	1.3 ± 0.14^c^

The Ca^2+^-iPLA_2_ activity was measured in homogenates of total spermatozoa from *Prdx6* ^−/−^ or WT mice treated with MJ33. Data are presented as mean ± S.E.M. Different letters in the same column denote significant differences (n = 3; *P* ≤ 0.05). ND, none detected.

### The absence of PRDX6 or the inhibition of Ca^2+^-iPLA_2_ activity of PRDX6 impaired fertilizing competence of mouse spermatozoa

**Figure 1:**
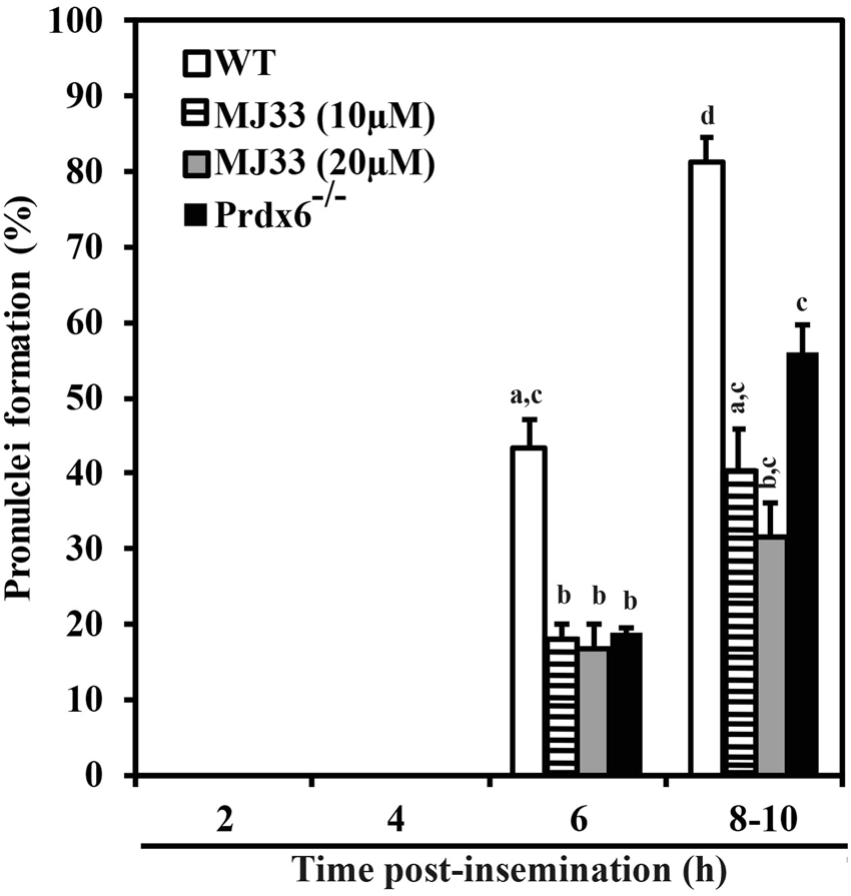
Deficiency of PRDX6 or inhibition of its Ca^2+^-iPLA_2_ activity negatively impacted pronuclear formation after IVF. Proportions of oocytes showing pronuclear formation at different times post-insemination (pi) with WT (treated or not with MJ33) and *Prdx6* ^−/−^ spermatozoa. 20–30 inseminated oocytes were evaluated in each group and time point. Data are presented as means ± S.E.M. Different letters denote significant differences (n = 3; *P* ≤ 0.05).

Fertilizing competence of C57 BL/6 J inbred and CD1 outbred mice spermatozoa was compared. The cleavage rates (24 h post-insemination (pi)) were significantly decreased when *Prdx6* ^−/−^ were compared to C57BL/6 J (WT) or CD1 spermatozoa were used in the *in vitro* fertilization studies (Table 2). Moreover, treatment of WT or CD1 spermatozoa with MJ33 during sperm capacitation significantly decreased the cleavage rates compared with those fertilized in the absence of the inhibitor. No blastocysts developed from the oocytes fertilized with *Prdx6* ^−/−^ spermatozoa. We observed a significant reduction of blastocyst production when oocytes were fertilized with WT or CD1 spermatozoa capacitated in the presence of MJ33 compared to the untreated controls. In order to determine if these perturbations in the cleavage and embryo development are due to defects in the fertilizing ability of the spermatozoa, we examined the formation of male and female pronuclei at 2, 4, 6 and 8–10 h pi. We found that at 2 and 4 h p.i, oocytes that were fertilized with *Prdx6* ^−/−^ spermatozoa or with WT spermatozoa capacitated in the presence or absence of MJ33 did not show any pronuclear formation (Fig. 1). At 6 h pi, 43.5% of oocytes that were fertilized with non-treated WT spermatozoa showed male and female pronuclei. This percentage was significantly higher than those inseminated with *Prdx6* ^−/−^ spermatozoa or with MJ33-treated WT spermatozoa. At 8–10 h pi, oocytes fertilized with WT spermatozoa had the highest percentage of pronculei formation, whereas those inseminated with MJ33-treated or *Prdx6* ^−/−^ spermatozoa displayed a similar percentage of pronuclei formation as for those oocytes inseminated with WT spermatozoa and evaluated at 6 h pi (Fig. 1 and supplementary Fig. S2). These results indicate a delay in the formation of pronuclei in the oocytes that were fertilized with *Prdx6* ^−/−^ spermatozoa or with MJ33-treated WT spermatozoa. Moreover, the lack of the Ca^2+^-iPLA_2_ activity of PRDX6 impairs pronuclear formation, cleavage and subsequent embryo development after IVF.

**Table 2:** Defeciency of PRDX6 and/or inhibition of PRDX6 Ca^2+^-iPLA_2_ activity impair fertilization and preimplantation embryo development in mice.

Mice	MJ33 (µM)	N Oocyte	Cleavage n (%)	Blastocyst n (%)
*Prdx6* ^−/−^	0	53	7 (13.2 ± 0.3)^a^	0 (0.0%)^a^
WT	0	38	25 (65.9 ± 2.3)^b^	8 (32.0 ± 0.7)^b^
	10	43	12 (27.9 ± 0.6)^a,c^	2 (16.7 ± 0.3)^c^
	20	41	7 (17.1 ± 1.2)^a^	1 (14.3 ± 0.3)^c^
CD1	0	73	58 (79.5 ± 2.1)^b^	34 (58.6 ± 1.9)^b^
	10	63	35 (55.6 ± 2.8)^b,d^	15 (42.9 ± 1.7)^d^
	20	48	20 (41.7 ± 2.0)^c,d^	8 (40.0 ± 0.8)^d^

Cleavage (24 h pi) and blastocyst development (4 days pi) after IVF of ovulated CD1 oocytes with *Prdx6* ^−/−^, WT or CD1 spermatozoa treated with MJ33 during sperm capacitation. Data are presented as mean ± S.E.M. Different letters in the same column denote significant differences (n = 3; *P* ≤ 0.05).

### The absence of PRDX6 or the inhibition of PRDX6 Ca^2+^-iPLA_2_ activity impaired sperm motility and viability

**Figure 2:**
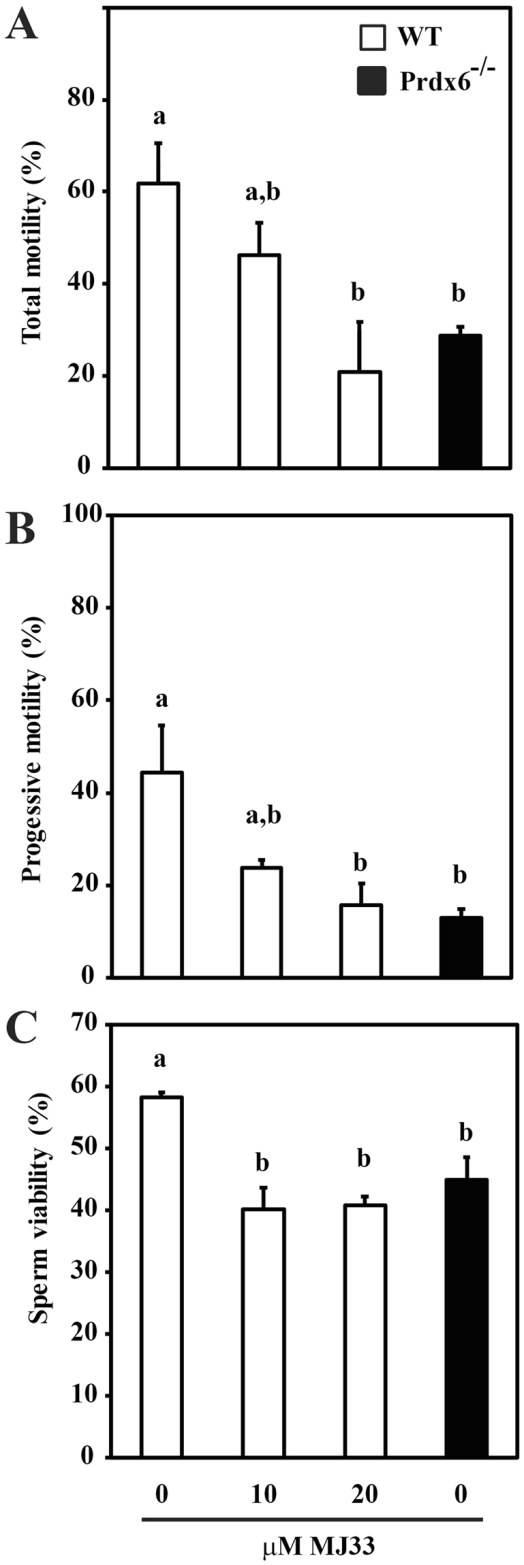
Lack of PRDX6 or inhibition of its Ca^2+^-iPLA_2_ activity reduced sperm motility and viability in WT and *Prdx6* ^−/−^ mice. Percentages of total (**A**), progressive motility (**B**), and (**C**) viability in MJ33-treated WT and in *Prdx6* ^−/−^ spermatozoa are presented as mean ± S.E.M. Different letters denote significant differences (n = 4–6; *P* ≤ 0.05).

Total and progressive motility were lower in *Prdx6* ^−/−^ spermatozoa than in WT controls (Fig. 2A and B). Incubation of WT spermatozoa with MJ33 reduced progressive motility in a dose-dependent manner. The percentage of viable cells was significantly lower in *Prdx6* ^−/−^ compared to WT controls (Fig. 2C). Treatment of WT spermatozoa with MJ33 reduced the percentage of viable cells to similar levels as observed in *Prdx6* ^−/−^ spermatozoa. There were no differences in kinetic parameters when comparing WT (treated or not with MJ33) and *Prdx6* ^−/−^ spermatozoa (Supplementary Table S1).

### Lack of PRDX6 or inhibition of its Ca^2+^-iPLA_2_ activity increased the levels of lipid peroxidation and mitochondrial superoxide anion in spermatozoa

**Figure 3:**
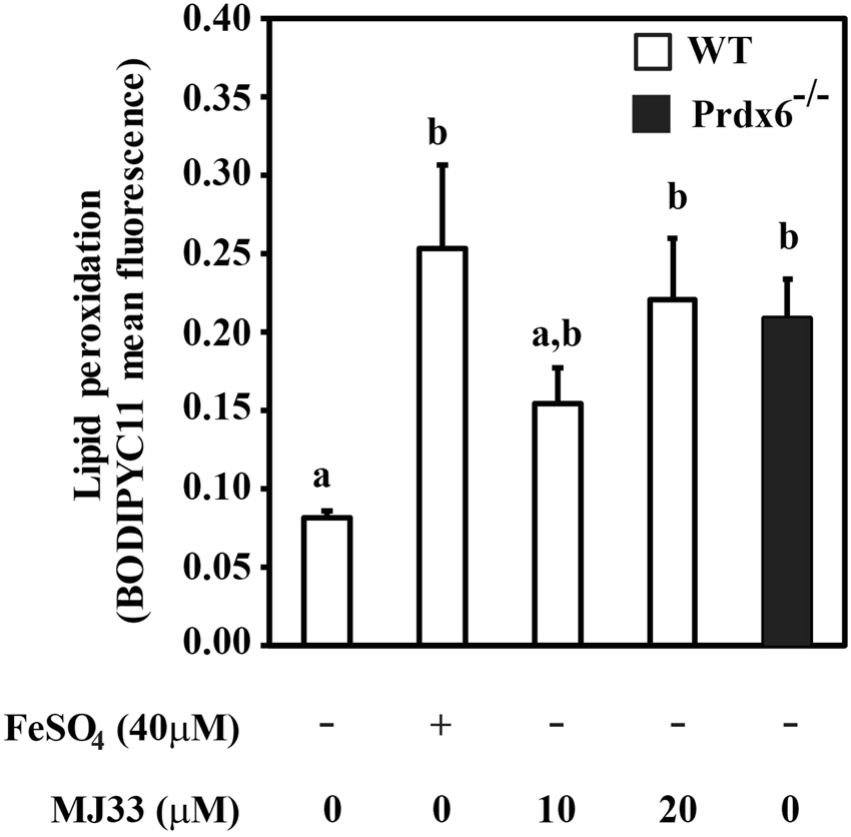
Lipid peroxidation levels are increased due to the absence of PRDX6 or the inhibition of its Ca^2+^-iPLA_2_ activity. BODIPY C11 mean fluorescence in MJ33-treated WT spermatozoa and in *Prdx6* ^−/−^ spermatozoa. WT spermatozoa treated with FeSO_4_ (40 μM) were used as positive controls. Different letters indicate significant differences (n = 4–7; *P* ≤ 0.05).

The levels of lipid peroxidation were significantly higher in *Prdx6* ^−/−^ spermatozoa as compared to WT controls (Fig. 3). Moreover, incubation of WT spermatozoa with MJ33 increased the levels of lipid peroxidation similar to those observed in *Prdx6* ^−/−^ or by treating spermatozoa with 40 µM FeSO_4_ (positive control).

**Figure 4:**
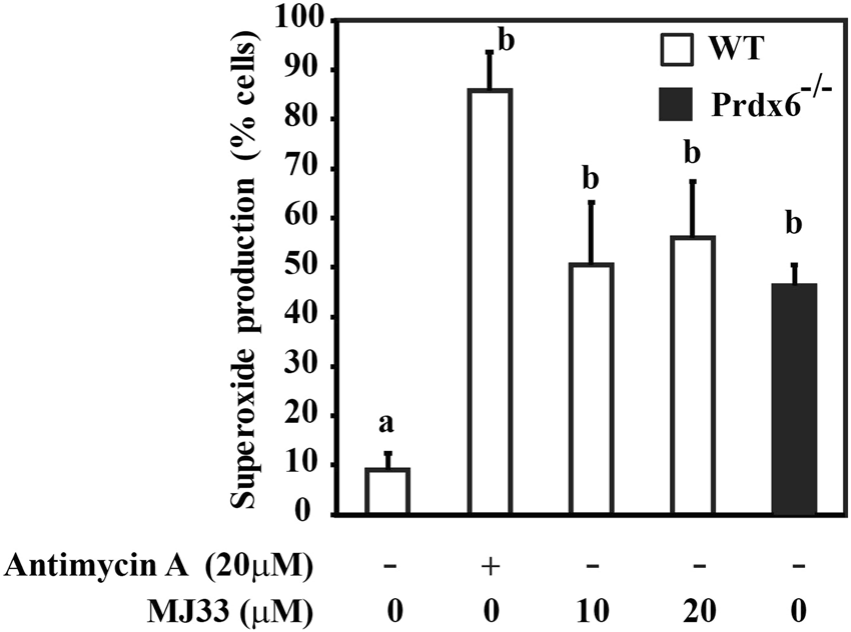
Levels of superoxide (O_2_^•−^) were increased due to the absence of PRDX6 or the inhibition of its Ca^2+^-iPLA_2_ activity in spermatozoa. Percentage of MJ33-treated WT and of *Prdx6* ^−/−^ spermatozoa with MitoSox red fluorescence (production O_2_^•−^). WT spermatozoa incubated in the presence of 20 µM Antimycin were used as positive controls. Different letters indicate significant differences (n = 4–10; *P* ≤ 0.05).

The percentage of viable cells that produce mitochondrial O_2_^•−^ was significantly higher in P*rdx6* ^−/−^ than in WT spermatozoa (Fig. 4). Incubation of WT spermatozoa with MJ33 significantly increased the level of O_2_^•−^ compared to the controls. The high levels of O_2_^•−^ seen in the mitochondria of *Prdx6* ^−/−^ spermatozoa and those of MJ33-treated WT spermatozoa indicate that the Ca^2+^-iPLA_2_ of PRDX6 is responsible for controlling the levels of O_2_^•−^.

### Lack of PRDX6 and the inhibition of Ca^2+^-iPLA_2_ activity of PRDX6 impaired sperm-zona binding and sperm-egg fusion

**Figure 5:**
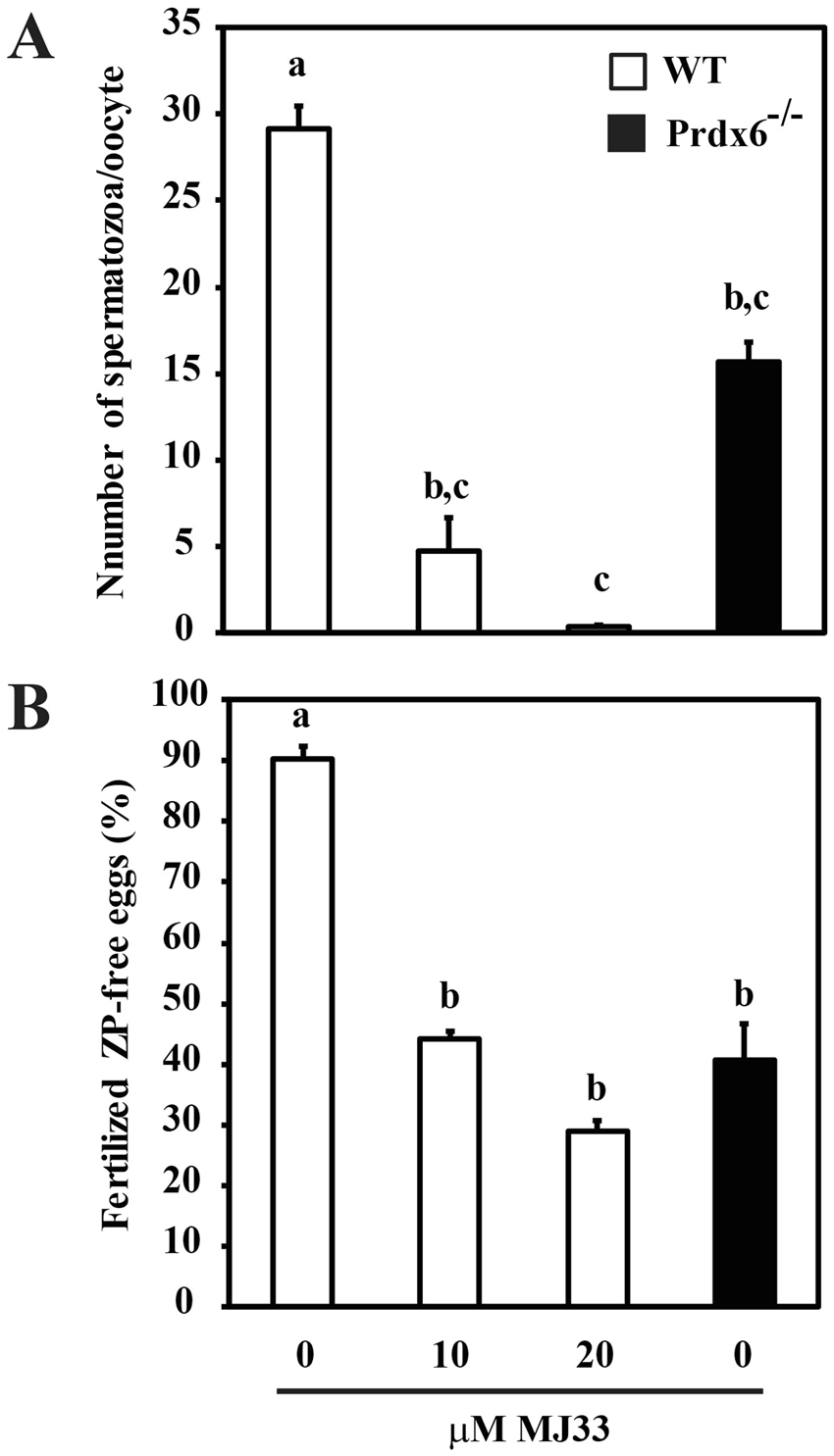
The absence of PRDX6 or the inhibition of its Ca^2+^-iPLA_2_ activity impaired the zona pellucida binding and fusion to the oolemma of *Prdx6* ^−/−^ or WT spermatozoa. (**A**) number of spermatozoa/oocyte following IVF of zona intact MII-oocytes with *Prdx6* ^−/−^ and MJ33-treated WT spermatozoa. (**B**) Percentage of fertilized oocytes using WT (treated or not with MJ33) or *Prdx6* ^−/−^ capacitated spermatozoa after IVF with zona-free ovulated CD1 oocytes. Data are presented as means ± S.E.M. Different letters denote significant differences (n = 3; *P* ≤ 0.05).

In order to determine whether the absence of PRDX6 impacts on the ability of the spermatozoa to bind to the zona pellucida (ZP), a necessary step prior to oocyte penetration by the sperm cell, we compared the zona-binding capacity of *Prdx6* ^−/−^ and WT spermatozoa in the presence or absence of PRDX6 inhibitor. We found that the number of *Prdx6* ^−/−^ spermatozoa/oocytes was significantly lower than WT controls (Fig. 5A). The incubation of WT spermatozoa with MJ33 resulted in a severe reduction in sperm-zona binding compared to control or, at high concentrations, even to *Prdx6* ^−/−^ spermatozoa.

To further elucidate the role of PRDX6 in the fertilization process, we evaluated sperm-fusion of both *Prdx6* ^−/−^ and WT spermatozoa previously capacitated in the presence or absence of MJ33. Treatment of WT spermatozoa with MJ33 affected their ability to fuse with the oolemma of ZP-free eggs (Fig. 5B). Indeed, these low numbers of fused spermatozoa were comparable to those seen in *Prdx6* ^−/−^ mice and significantly lower than those in WT controls. These results indicate that absence of PRDX6 or inhibition of its Ca^2+^-iPLA_2_ activity is associated with impaired sperm-egg recognition and egg fusion events.

### Spermatozoa lacking PRDX6 or treated with PRDX6 Ca^2+^-iPLA_2_ activity inhibitor have impaired ability to undergo the acrosome reaction

**Figure 6:**
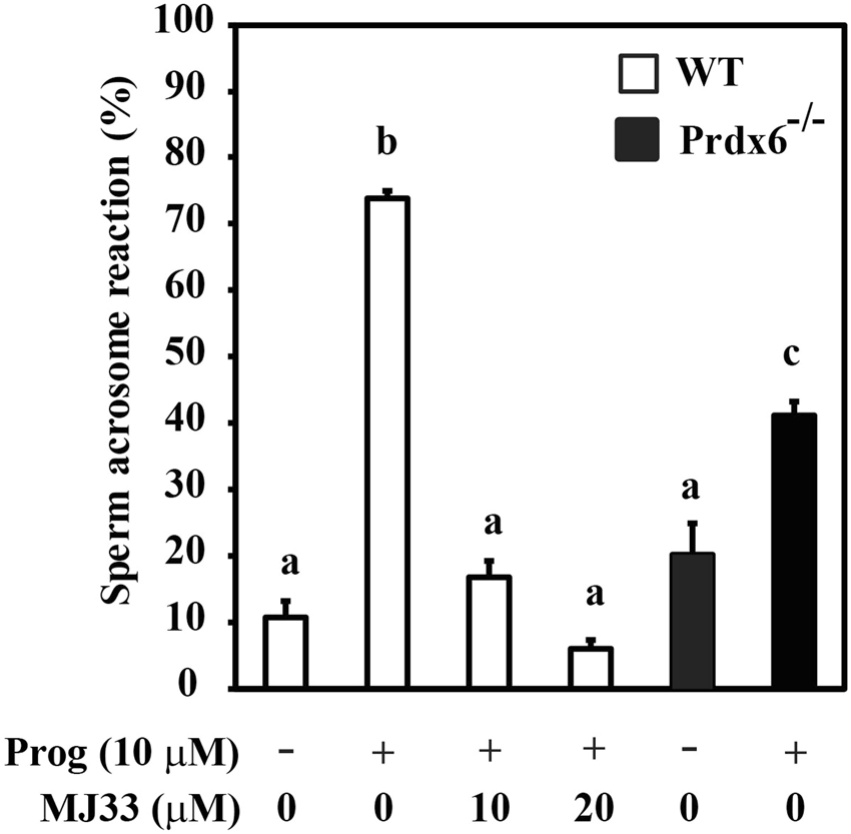
The absence of PRDX6 or the inhibition of its Ca^2+^-iPLA_2_ activity impaired acrosome reaction in capacitated spermatozoa. Percentage of acrosome reaction in WT (treated or not with MJ33) or *Prdx6* ^−/−^ capacitated incubated with progesterone (acrosome reaction inducer). Data are presented as means ± S.E.M. Different letters denote significant differences (n = 3; *P* ≤ 0.05).

The percentage acrosome reaction was lower in *Prdx6* ^−/−^ capacitated spermatozoa than WT controls (Fig. 6). Incubation of WT spermatozoa with MJ33 during sperm capacitation significantly reduced the percentage of acrosome reaction to comparable levels to those seen in the *Prdx6* ^−/−^ spermatozoa.

## Discussion

The present study demonstrates that inhibition of the PRDX6 Ca^2+^-iPLA_2_ activity by MJ33 is associated with severe perturbations in sperm functions that are associated with a reduction in the *in vitro* fertilizing ability of mouse spermatozoa. Although not yet studied in spermatozoa, PRDX6, through its Ca^2+^-iPLA_2_ as well as its phospholipid hydroperoxoidase activities is necessary to repair membranes that have been damaged oxidatively^40,41^; the integrity of these membranes in spermatozoa (plasma and inner and outer acrosome membranes) is essential to guarantee the proper recognition of the oocyte by the spermatozoon and assure the fusion of the sperm plasma membrane with the oolemma to allow the penetration of the oocyte and subsequent fertilization.

*Prdx6* ^−/−^ males produce lower numbers of litters and pups/male compared to WT controls and this abnormal reproductive outcome is associated with low chromatin quality and impaired motility of *Prdx6* ^−/−^ spermatozoa^38,39^. The amount of PRDX6 is low in spermatozoa from infertile men^27^. Moreover, the thiol oxidation status of this PRDX isoform is associated with reduced sperm motility and high levels of sperm DNA fragmentation in those infertile men. Altogether, these findings highlight the need for PRDX6 to protect mammalian spermatozoa against oxidative stress. Recently, we reported that inhibition of the Ca^2+^-iPLA_2_ activity of PRDX6 impaired human sperm capacitation, confirming the necessity of PRDX6 to assure male fertility^37^. Collectively, our current and previous findings support the critical role of PRDX6 in protecting sperm against oxidative stress to guarantee normal fertility.

In the present study, we further evaluated the effects of inhibiting PRDX6 in mice spermatozoa on pronuclear formation, cleavage rates and embryo development following *in vitro* fertilization with ovulated mature oocytes (Fig. 1 and Table 2). The inhibition of the Ca^2+^-iPLA_2_ of PRDX6 by MJ33 in spermatozoa impaired pronuclear formation, cleavage rates and blastocyst development, indicating the need for this activity to assure normal fertilization and production and the early development of embryos. The fact that *Prdx6* ^−/−^ spermatozoa promote a severe reduction in cleavage and no blastocyst development indicates that these spermatozoa are incapable of generating viable embryos *in vitro*, compatible with their increased susceptibility to oxidative stress. These findings may indicate that failure of sperm from infertile men to fertilize oocytes using assisted reproductive technologies, may reside in the inactivation of PRDX6 of their spermatozoa. Based on the present results, we propose a requirement for PRDX6 to protect sperm against oxidative stress, a known culprit that negatively impacts the preimplantation embryonic development^42–44^.

The gametes of inbred C57BL/6 J mice are much more vulnerable to manipulations *in vitro* than those of CD1 outbred mice^45^. The evidence from the present study that the inhibition of Ca^2+^-iPLA_2_ of PRDX6 by MJ33 in CD1 mice spermatozoa impaired the cleavage and blastocyst rates highlights the importance of PRDX6 in protecting the sperm against oxidative stress even in spermatozoa known to perform very well under *in vitro* conditions. The defects in the cleavage and blastocyst development following IVF of matured oocytes with spermatozoa treated with the PRDX6 inhibitor could be ascribed to various perturbations in some biological sperm functions such as capacitation that are known to be essential for successful completion of fertilization and embryo development. Recently, we reported that MJ33 prevented sperm capacitation and its associated actin polymerization in human spermatozoa^37^.

Abnormal sperm motility is strongly correlated with *in vitro* fertilization failure^46^ and it is well documented that increased levels of ROS production are associated with a reduction of sperm motility^14,47–50^. Interestingly here, the inhibition of PRDX6 by MJ33 is associated with a decrease in both total and progressive motility in WT mice spermatozoa. These findings, along with the decreased sperm motility observed in *Prdx6* ^−/−^ mice compared to WT controls (Fig. 2A and B), indicate a major protective effect of PRDX6 to maintain intact motility machinery. *Prdx6* ^−/−^ spermatozoa are highly susceptible to oxidative stress displaying oxidative damage such as protein carbonylation and S-glutathionylation with a significant reduction in their motility^14,39^. Tubulin, the major component of the sperm flagellum, is oxidized by high levels of hydrogen peroxide (H_2_O_2_)^51^, thus explaining the impairment of sperm motility linked with the oxidative stress observed in the current and our previous studies^14,38,39^. Furthermore, these results support our previous findings indicating that infertile men with low amounts of PRDX6 or having high levels of thiol oxidation of PRDX6 in their spermatozoa displayed reduced motility^27^.

ROS have long been implicated in the lipid peroxidation due to the presence of high amounts of polyunsaturated fatty acids in membranes of mammalian spermatozoa. Unsaturated lipids are highly susceptible to peroxidation and excess ROS have a negative influence on sperm motility and fertility^52,53^. *Prdx6* ^−/−^ spermatozoa have higher levels of lipid peroxidation than those from WT controls (Fig. 3) and the inhibition of Ca^2+^-iPLA_2_ of PRDX6 by MJ33 in WT spermatozoa increased the levels of lipid peroxidation compared with non-treated controls. Altogether these results suggest the need of PRDX6 Ca^2+^-iPLA_2_ in addition to its peroxidase activity for the protection of spermatozoa against lipid peroxidation. The protection of sperm lipid membranes by PRDX6 was recognized in other mammalian species; we recently showed that incubation of human spermatozoa with MJ33 increased the levels of lipid peroxidation and the values increased further in capacitated spermatozoa, a process known to be associated with the production of low levels of ROS^26,37^. In mice, we also observed higher levels of lipid peroxidation (measured by the TBARS assay) in *Prdx6* ^−/−^ spermatozoa compared to their respective WT controls that were exacerbated by tert-BHP treatment^39^. Furthermore, spermatozoa from rats treated with tert-BHP have increased levels of lipid peroxidation with oxidatively inactivated PRDXs, particularly PRDX6^54^. Thus, these findings indicate that the antioxidant protection exerted by PRDX6 in spermatozoa is a common phenomenon occurring in different mammalian species.

Peroxidation of the unsaturated fatty acids within the sperm plasma membrane is associated with loss of fluidity, structure, and function of the membranes that is negatively correlated with sperm competence^55^. The indication that sperm viability is lower in *Prdx6* ^−/−^ than in the WT spermatozoa and that the inhibition of the Ca^2+^-iPLA_2_ activity of PRDX6 by MJ33 in WT spermatozoa reduced viability (Fig. 2C), confirms the essential role of PRDX6 as the main enzyme responsible for removing the lipid peroxides to maintain a healthy plasma membrane in the spermatozoon.

It is well known that mitochondria are an important source of ROS in the spermatozoa by the formation of O_2_^•−^ in the electron transport chain^20^. Excessive production of mitochondrial ROS (mROS), such as O_2_^•−^ and H_2_O_2_ (generated by the spontaneous or enzymatic dismutation of O_2_^•−^), in the sperm has been reported to result in membrane peroxidation and loss of motility^20^. The absence of PRDX6 in spermatozoa is associated with increased levels of O_2_^•−^ (Fig. 4) and the inhibition of the Ca^2+^-iPLA_2_ activity of PRDX6 by MJ33 significantly increased the levels of O_2_^•−^ generation by mitochondria in WT spermatozoa to levels comparable with those seen in spermatozoa treated with antimycin A (positive control, Fig. 4). Altogether, these findings strongly support the role of PRDX6 as the primary antioxidant in mouse spermatozoa.

A unique function of the spermatozoon is its interaction with the oocyte to fertilize it and deliver the paternal genome to produce the zygote. Thus, the spermatozoon is required to undergo capacitation, a complex process that includes biochemical, morphological and dynamic changes^26,56^, in order to be competent for oocyte fertilization. Fully capacitated spermatozoa undergo the acrosome reaction, the exocytotic event that will release hydrolytic enzymes to allow the spermatozoon to penetrate the ZP^57^. *Prdx6* ^−/−^^39^ and WT spermatozoa treated with MJ33 (this study) have impaired capacitation since they failed to undergo the acrosome reaction (Fig. 6). The negative impact of PRDX6 inhibition on sperm capacitation and acrosome reaction may be ascribed to the possible oxidation of the key proteins involved in these processes. We recently demonstrated that oxidative stress impairs human sperm capacitation, even when ROS levels are low enough not to impair sperm motility^14^ and that MJ33 also inhibited this process^37^. Thus, PRDXs are important participants in the regulation of sperm ROS levels to allow capacitation without its increase to toxic levels.

As mentioned above, the recognition and binding of the spermatozoon to the ZP is a crucial step in the fertilization process. Our results suggest that inhibition of Ca^2+^-iPLA_2_ of PRDX6 severely impaired the ability of the spermatozoa to recognize and bind to the ZP. Because the sperm plasma membrane plays a critical role in sperm-oocyte recognition, adhesion, and fusion with the oolemma for fertilization, we propose that the perturbations of the sperm plasma membrane due to the inhibition of or the lack of PRDX6 lowers the ability of the spermatozoon to recognize and bind to the zona pellucida, leading to infertility. Recently, it has been shown that oxidative stress has a negative impact on sperm-egg interaction and this inhibition of ZP binding is associated with impaired surface expression of ZP-receptor arylsulphatase A^58^. These results suggest that PRDX6 is required for maintaining sperm membrane integrity to assure normal binding to the ZP. The molecular mechanism by which MJ33-treated spermatozoa are less capable than controls or even *Prdx6* ^−/−^ spermatozoa to bind to ZP is unknown. MJ33 mimics the transition state of a phospholipid and binds competitively to the PLA_2_ prote^59^. It is possible that, due to its lipid nature, MJ33 is incorporated into to the sperm plasma membrane and thus may alter the lipid rafts involved in the sperm-ZP binding^60,61^. The plasma membrane that interacts with the ZP has a different composition of lipids and proteins from that of the equatorial segment involved in the fusion of the spermatozoon with the oolema^62^. Thus, MJ33 not only inhibits PRDX6 Ca^2+^-iPLA_2_ activity thereby promoting oxidative damage of sperm proteins involved in the recognition of the ZP, but also modifies the plasma membrane structure involved in this interaction. Both actions of MJ33 result in the inhibition of the binding to the ZP by WT spermatozoa.

Any defect of the plasma membrane that could occur as a result of oxidative stress will negatively impact the proteins involved in sperm-egg fusion. Plasma membrane sperm protein IZUMO1 is recognized as an essential protein for sperm-egg fusion since IZUMO1 knockout males are infertile^63–65^. The disulfide isomerase ERp57 is important for sperm-egg fusion^66^ and the blocking of membrane protein thiol groups with a membrane-impermeable thiol-reactive reagent, 4-acetamido-40-((iodoacetyl)amino)stilbene-2,20-disulfonic acid, resulted in a decrease in the number of sperm fused with the oocytes^64^. Then, it is possible that the impairment of spermatozoa from *Prdx6* ^−/−^ mice or MJ33-treated WT spermatozoa is due to the oxidized plasma membrane thiols of essential proteins such as IZUMO1. These oxidized thiols are no longer able to participate in the formation of disulfide groups to complete the conformational changes of IZUMO1 to trigger the fusogenic process after its binding with JUNO (its female binding partner) and allow the fertilization of the oocyte. These results confirm the critical role of PRDXs in protecting those sperm surface proteins that are known to be essential for sperm-egg fusion against oxidation. The lower fusion ability of *Prdx6* ^−/−^ and WT spermatozoa treated with MJ33 may be ascribed to the high levels of lipid peroxidation, the increase in membrane fluidity and defects in acrosome reaction that we have reported here.

In conclusion, the inhibition of PRDX6 Ca^2+^-iPLA_2_ activity results in an oxidative stress and the inhibited removal of lipid peroxides that are associated with abnormal sperm functions and compromised fertilizing ability. PRDX6 is necessary to protect the sperm membranes for sperm-zona binding and fusion with the oolemma prior to fertilization. Our findings, along with our previous results on the role of PRDXs in protecting human spermatozoa against oxidative stress^27,37^, provide strong evidence for the crucial role of PRDXs, particularly PRDX6, in male reproduction.

## Methods

### Reagents

Minimum Essential Medium (MEM) Alpha medium, 4,4-difluoro-5-(4-phenyl-1,3-butadienyl)-4-bora-3a,4a-diaza-s-indacene-3-undecanoic acid (BODIPY 581/591 C11), MitoSox Red and Sytox Green were purchased from Life Technologies (Burlington, ON, Canada). Potassium-supplemented optimized medium (KSOM) was bought from Millipore (Etobicoke, ON, Canada). 1-Hexadecyl-3-(trifluoroethyl)-*sn*-glycero-2-phosphomethanol lithium (MJ33), Bouin fixative, hematoxylin and eosin were purchased from Sigma-Aldrich (St. Louis, MO, USA). Other chemicals used were of at least reagent grade.

### Animals

All experiments were carried out according to the Guide to the Care and Use of Experimental Animals issued by the Canadian Council on Animal Care and with approval from the Animal Care Committee of McGill University. The *Prdx6* ^−/−^ mouse model was developed by Dr. YS Ho (Wayne State University) in collaboration with the laboratory of Dr. Aron Fisher at the University of Pennsylvania^67^; the mice were then backcrossed to the C57BL/6 with > 99.9% genetic identity as determined by microsatellite analysis done by the Jackson Laboratory^68^. A colony of these mice was established at the Research Institute, McGill University Health Centre^38,39^. C57BL/6 (WT), CD1 males and CD1 females were purchased from Charles River (Montreal, QC, Canada). All animals were maintained under pathogen-free conditions with a humidity range of 30%–60%, a temperature range of 21 °C−24 °C, a light cycle of 12 L:12D, and ad libitum food and water supply.

### Testis collection and histology

WT or *Prdx6* ^−/−^ male mice were euthanized and testes were dissected, weighed and fixed immediately with Bouin fixative for 24 hours. The tissue was processed using routine paraffin-embedding methods. Paraffinized-tissue blocks were sectioned at 5 μm thickness and stained with haematoxylin and eosin (H&E).

### Sperm preparation

Sperm were collected from cauda epididymides of *Prdx6* ^−/−^ and WT mice at 8-wk-old by poking them with a 27-gauge needle and letting them disperse in 500-µL of Biggers, Whitten and Whittingham medium (BWW, pH 7.4) composed of 91.5 mM NaCl, 4.6 mM KCl, 1.7 mM CaCl_2_, 1.2 mM KH_2_ PO_4_, 1.2 mM MgSO_4_, 25 mM NaHCO_3_, 5.6 mM D-glucose, 0.27 mM sodium pyruvate, 44 mM sodium lactate, and 20 mM Hepes^69^ for 10 min at 37 °C. Sperm concentration was determined with a hemocytometer and then adjusted to 5 × 10^6^ cells/ml in BWW^38^.

### PRDX6 Ca^2+^-iPLA_2_ activity assay

Ca^2+^-iPLA_2_ activity of total spermatozoa homogenate was measured as previously described^70^. Briefly, 8 × 10^6^ spermatozoa were incubated in BWW with 0, 10, or 20 µM MJ33 for 2 h at 37 °C. Then, sperm samples were lysed in RIPA buffer (10 mM Tris-Cl (pH 7.2), 5 mM EDTA, 1% Triton X-100, 0.1% sodium deoxycholate, 0.1% SDS, 150 mM NaCl) and sonicated three times for 15 sec at 30% output with a Sonic Vibracell (Sonics and Materials, Inc., Newtown, CT, USA). The total amount of protein was determined by the Bradford method. For assay, ~0.5 mg of protein/ml was incubated with mixed unilamellar liposomes containing 50% dipalmitoyl phosphatidylcholine (DPPC) labeled with [*methyl*-^3^H]choline, 25% egg phosphatidylcholine, 10% phosphatidylclycerol, and 15% cholesterol in pH 4.0 buffer (40 mM Sodium acetate, 5 mM EDTA) for 60 min at 37 °C. Lipid classes with appropriate controls were separated by thin layer chromatography. Total phospholipase activity was calculated from the decrease in radioactivity (d.p.m.) recovered in DPPC and expressed as nmol/h per mg of protein.

### Determination of sperm motility and viability

Spermatozoa from *Prdx6* ^−/−^ and WT mice were incubated with 0, 10, or 20 µM MJ33 (an inhibitor of the Ca^2+^-iPLA_2_ activity of PRDX6^71^) for 2 h at 37 °C. Following the incubation, sperm total and progressive motility were evaluated by using a computer-assisted sperm analysis system (CASA) with Sperm Vision HR software version 1.01 (Minitube, Ingersoll, ON, Canada). A total of 200 spermatozoa were examined for each sample to determine the percentages of sperm total and progressive motility. Sperm samples were centrifuged for 5 min at 650 × g at 20 °C and then washed and incubated with 0.05 µM Sytox Green in Hanks’ balanced salt solution (HBSS) for 15 min at 37 °C in the dark in order to determine viability. The Sytox Green (green) fluorescence, an indicator for dead cells, was measured using a MACSQuant Analyzer flow cytometer (Miltenyi Biotec, Inc., Auburn, CA, USA) equipped with an argon laser (488 nm) at FL-1 (530/30 nm band pass filter). A minimum of 10,000 spermatozoa were analyzed for each sample.

### Determination of lipid peroxidation

Levels of lipid peroxidation were determined by flow cytometry using BODIPY 581/591 C11 probe according to the method described by Aitken *et al*.^72^ with modifications. Spermatozoa collected from *Prdx6* ^−/−^ and WT mice were incubated with or without MJ33 in BWW medium at 37 °C for 2 h. Spermatozoa were then centrifuged for 5 min at 650 × g at 20 °C and incubated with 5 µM BODIPY 581/591 C11 in HBSS for 30 min at 37 °C in dark. A positive control was prepared by incubating a sperm aliquot with 40 µM ferrous sulfate (FeSO_4_) for 2 h at 37 °C. A minimum of 10,000 spermatozoa were analyzed for each sample using a MACSQuant Analyzer flow cytometer (Miltenyi Biotec, Inc., Auburn, CA, USA) equipped with an argon laser (488 nm) and 585/625 nm filter. Levels of lipid peroxidation were expressed as relative intensity of green fluorescence/red + green fluorescence.

### Determination of mitochondrial superoxide anion

Mitochondrial superoxide anion (O_2_^•−^) generation was detected by flow cytometry using MitoSOX Red, a specific fluorescent probe for the O_2_^•−^ produced by mitochondria, as described previously^20^. Following incubation of *Prdx6* ^−/−^ and WT spermatozoa for 2 h with or without MJ33 in BWW medium, MitoSOX Red was added to the sperm aliquots at 2 µM final concentration. A positive control was obtained by incubating a sperm aliquot with 20 µM antimycin A for 2 h at 37 °C. Following incubation with MitoSox Red for 15 min at 37 °C in dark, spermatozoa were centrifuged at 650 × g for 5 min and then washed with HBSS. After centrifugation, spermatozoa were incubated with Sytox Blue (0.1 µM final concentration) to determine the percentage of viable cells. The MitoSOX Red and Sytox Blue fluorescences were measured using a MACSQuant Analyzer flow cytometer (Miltenyi Biotec, Inc., Auburn, CA, USA) equipped with a blue (488 nm) and violet (405 nm) lasers. A minimum of 10,000 events were analyzed for each sample.

### *In vitro* fertilization (IVF) and embryo culture

*In vitro* fertilization and embryo culture were conducted as described previously^45^. Briefly, *in vivo* ovulated cumulus-oocyte complexes (COCs) were collected from superovulated 8-to 12-wk-old CD1 females 14–15 h post-human chorionic gonadotropin (hCG) injection. In order to test the effect of PRDX6 inhibition on fertilizing ability and embryo development, spermatozoa collected from 8- to 10-wk-old *Prdx6* ^−/−^, WT and CD1 males were incubated with MJ33 (10 or 20 µM) in MEM-alpha medium supplemented with 0.9% BSA for 60 min at 37 °C with 5% CO_2_ in a humidified incubator for sperm capacitation. For IVF, COCs were inseminated with capacitated spermatozoa at 1 × 10^6^ cells/ml, the gametes were co-incubated together in IVF medium (MEM- alpha supplemented with 0.4% BSA) for 4–5 h at 37 °C with 5% CO_2_ in a humidified atmosphere. Inseminated oocytes were then thoroughly washed to remove adhered spermatozoa and cumulus cells by repeating pipetting. The fertilized eggs were cultured in modified KSOMaa (potassium simplex optimization medium with amino acids) for up to 4 days (Day 0 of insemination) at 37 °C under 5% CO_2_ in humidified atmosphere. The numbers of oocytes that developed to the two-cell and blastocyst embryo stages were recorded at 24 h and 4 days post-insemination (pi), respectively^45^.

### Evaluation of pronuclei formation

Detection of pronuclear formation after IVF was performed as previously described^73^ with modifications. Briefly, at 2, 4, 6 or 8–10 h pi, oocytes were washed carefully in M2 medium and then fixed in 2% (v/v) paraformaldehyde (PFA) in M2 for 20 min. After washing three times in M2 medium, oocytes were stained with Hoechst 33342 (10 µg/ml) for 10 min. Then, oocytes were transferred to a small drop of Vectashieldmounting medium containing 4,6-diamidino-2-phenylindole (DAPI, Vector laboratories Inc., Burlingame, USA.) on a microscope slides. The slides were examined under an automated fluorescence microscope system fluorescence microscope (Leica, DMR6000B, Germany). The number of oocytes showing male and female pronuclei in the cytoplasm was recorded.

### Sperm-Zona Pellucida Binding Assay

*In vivo* ovulated COCs collected from superovulated CD1 female mice at 8–12 wk old were denuded of cumulus cells by using 300 IU/ml hyaluronidase in the M2 medium. Completely denuded oocytes were *in vitro* fertilized by capacitated *Prdx6* ^−/−^ or WT spermatozoa capacitated *in vitro* in the presence or absence of MJ33. Sperm and oocytes were incubated together for 1 h at 37 °C under 5% CO_2_ in humidified atmosphere. Then, inseminated oocytes were washed three times with M2 medium and fixed in 2% paraformaldehyde and stained with 10 µg/ml Hoechst 33342. Bound sperm/oocyte was counted as the number of sperm heads found attached to zona pellucida (ZP) by an inverted microscope equipped with epifluorescence optics (Leica, DMIRB, Germany).

### Zona-free fusion assay

Sperm fusion with ZP-free eggs was performed as reported previously^74^ with minor modifications. Briefly, after removing cumulus cells from ovulated CD1 COCs, denuded oocytes were incubated with an acid Tyrode solution for 20–30 sec to dissolve the ZP. ZP-free eggs were inseminated with capacitated *Prdx6* ^−/−^ or WT spermatozoa treated with MJ33 for 1 h. Following 4 h gametes co-incubation, oocytes were washed with M2 medium to remove loosely adhered sperm. The oocytes were then fixed in 2% paraformaldehyde, stained with 10 µg/ml Hoechst 33342 and examined under an inverted microscope equipped with epifluorescence optics (Leica, DMIRB, Germany) to detect the presence of decondensing sperm heads in the ooplasm.

### Evaluation of acrosome reaction (AR)

Spermatozoa from *Prdx6* ^−/−^ or WT mice were collected as described above and then incubated in BWW medium supplemented with 4 mg/ml BSA and 20 mM NaHCO_3_^75^ as capacitating agents for 60 min at 37 °C. Only for WT spermatozoa, MJ33 (10 or 20 µM) was added to the capacitation medium. Spermatozoa were then centrifuged at 650 × g for 5 min and then resuspended in BWW with 10 µM progesterone for 30 min to induce acrosome reaction^39^. Spermatozoa that were incubated in BWW without any capacitating agents and/or without progesterone were set as negative controls. After incubation, sperm were centrifuged and fixed with 100% ethanol for 5 min. 20 µl of the sperm suspension was smeared on a glass slide and air dried. The acrosome status was detected by staining the spermatozoa with 30 µg/ml of fluorescein isothiocynate (FITC)-labeled *Pisum sativum* agglutinin (FITC-PSA) for 20 min^39^. After washing the slides with distilled water and air dried, a drop of 1,4-diazabicyclo[2.2.2]octane (DABCO) was added to each slide, and they were sealed with coverslips. The slides were examined under an epifluorescence microscope (Axiophot; Zeiss, Oberkochen, Germany) at 1,000 magnification to determine the status of the acrosome. A total of 200 spermatozoa per duplicate were analyzed to determine the presence or absence of acrosomes. Values were presented as percentage of spermatozoa without acrosome.

### Statistical analysis

Data are presented as means ± SEM; statistical differences between group means were determined using ANOVA and Bonferroni or Tukey test and Kruskal-Wallis test as appropriate. Chi-squared test was used to evaluate the differences in cleavage and embryo development rates between the groups. Statistical analysis was done by using Sigma Systat 13 (Systat Software Inc., San Jose, CA, USA). Differences among samples were considered to be significant when the P value is ≤0.05.

## Electronic supplementary material

Supplementary information
