# Supplementary material for: Deficiency of peroxiredoxin 6 or inhibition of its phospholipase A2 activity impair the in vitro sperm fertilizing competence in mice
Source: Sci Rep. 2017 Oct 11;7:12994. doi: 10.1038/s41598-017-13411-2 (PMC5636886; doi:10.1038/s41598-017-13411-2)
Supplement: Supplementary file 1 — Supplementary information [file 41598_2017_13411_MOESM1_ESM.pdf]

Deficiency of peroxiredoxin 6 or inhibition of its phospholipase A_2 activity impair the *in vitro* sperm fertilizing competence in mice

Adel R. Moawad[1,2,4], Maria C. Fernandez[1,2], Eleonora Scarlata[1,2], Chandra Dodia[5,6], Sheldon I. Feinstein[5,6], Aron B. Fisher[5,6] and Cristian O'Flaherty[1,2,3,7]

The Research Institute of the McGill University Health Centre[1] and Departments of Surgery (Urology Division)[2], Pharmacology and Therapeutics[3], McGill University, Montréal, Québec, Canada. Department of Theriogenology[4], Faculty of Veterinary Medicine, Cairo University, Giza, Egypt. Institute for Environmental Medicine[5], Department of Physiology[6], Perelman School of Medicine, University of Pennsylvania, Philadelphia, PA, USA

Supplementary Table S1: Sperm dynamics in *Prdx6*$^{-/-}$ and WT spermatozoa treated with PRDXs inhibitors.

Mice	MJ33 Concentration (μM)	DCL	DAP	DSL	VCL	VAP	VSL	LIN	STR	WOB	BCF	ALH	AOC
Prdx6$^{-/-}$	0	85.7±8.1	51.8±2.1	32.8±1.4	204.4±17.4	126.8±6.1	81.6±4.4	0.4±0.0	0.6±0.0	0.6±0.0	19.2±1.2	11.4±0.5	16.2±0.8
WT	0	103.1±9.6	57.0±4.5	32.0±1.6	249.5±22.9	140.7±11.7	80.9±4.6	0.3±0.0	0.6±0.0	0.6±0.0	19.9±1.8	10.9±1.0	20.1±1.6
	10	77.4±6.3	48.3±2.9	31.4±1.1	186.2±13.4	118.4±6.5	78.4±2.5	0.4±0.0	0.7±0.0	0.6±0.0	13.7±1.3	11.1±0.5	17.6±0.5
	20	76.1±9.7	45.3±3.0	32.7±2.8	191.2±16.8	119.1±10.2	89.6±14.3	0.5±0.1	0.7±0.1	0.6±0.0	25.6±10.9	11.9±1.3	16.3±1.5

Sperm dynamics such as distance curved line (DCL), distance average path (DAP), distance straightline (DSL), curvilinear velocity (VCL), average path velocity (VAP), straight line velocity (VSL), linearity (LIN), straightness (VSL/VAP; STR), WOB (VAP/VCL; wobble), beat cross frequency (BCF), amplitude of lateral head (ALH), orientation of head (AOC) were evaluated by using CASA. DCL, DAP, VCL, and VAP were impaired in *Prdx6*$^{-/-}$ spermatozoa and after incubating WT or *Prdx6*$^{-/-}$ spermatozoa with MJ33, but the differences did not reach to significant effect. Data are presented as mean ± S.E.M, n= 4-6.

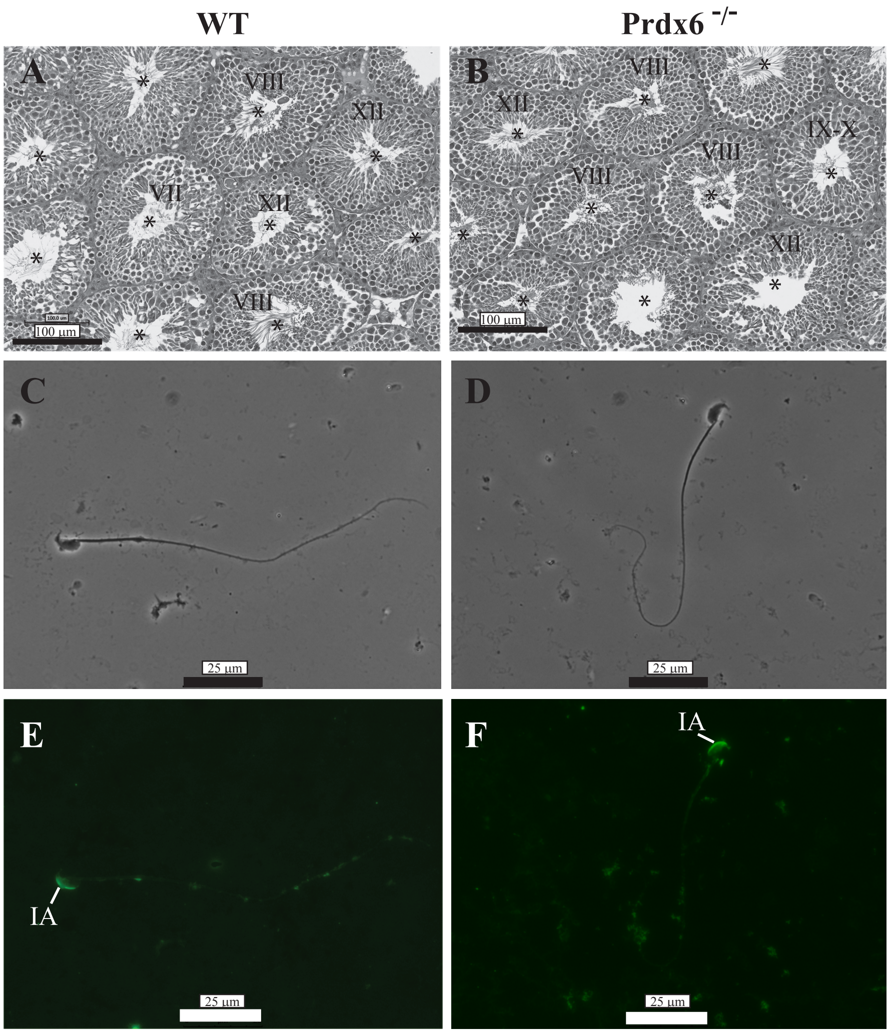

Supplementary Figure S1. Histology of testis and sperm morphology in WT and Prdx6$^{-/-}$ males. Hematoxylin-eosin-stained sections of WT (A) and Prdx6$^{-/-}$ (B) testis. *Indicates the presence of elongated spermatid or spermatozoa in the seminiferous tubules lumen. Roman numbers indicate the stage of spermatogenesis. Bar = 100 µm. Spermatozoa from WT (C and E) and Prdx6$^{-/-}$ (D and F) males. Intact acrosome (IA) were labeled with Pissum sativum conjugated with FITC (E and F, see methods). Bar = 25 µm.

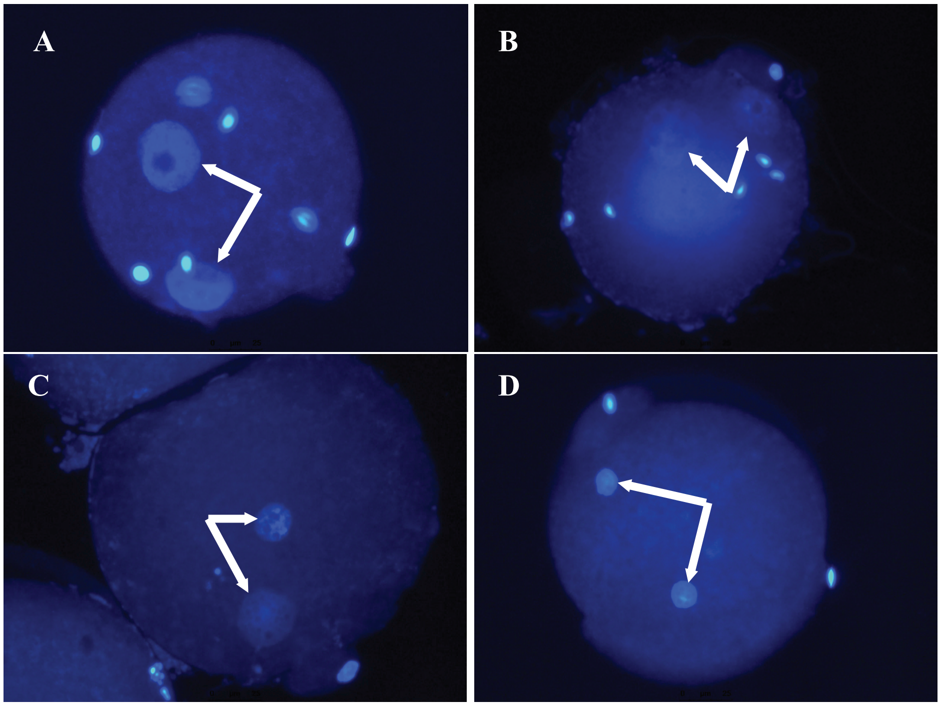

Supplementary Figure S2. Male and female pronuclei (white arrows) at 10 h post-insemination of ovulated CD1-oocytes with (A) WT spermatozoa, (B) Prdx6$^{-/-}$ spermatozoa, (C) WT spermatozoa treated with 10 µM MJ33, (D) WT spermatozoa treated with 20 µM MJ33. The oocytes were stained with DAPI and examined under fluorescence microscope at 400 magnifications.
